# Differences in Corticoreticulospinal Tract Injuries According to Whiplash in Mild Traumatic Brain Injury Patients

**DOI:** 10.3389/fneur.2019.01199

**Published:** 2019-11-29

**Authors:** Sung Ho Jang, Sang Seok Yeo, Jung Won Kwon, Young Hyeon Kwon

**Affiliations:** ^1^Department of Physical Medicine and Rehabilitation, College of Medicine, Yeungnam University, Gyeongsan, South Korea; ^2^Department of Physical Therapy, College of Health Sciences, Dankook University, Seoul, South Korea

**Keywords:** corticoreticulospinal tract, diffusion tensor tractography, balance error scoring system, postural control ability, whiplash injury, mild traumatic brain injury

## Abstract

**Background:** This study investigated differences in postural control ability (PCA) and corticoreticulospinal tract (CRT) injury severity according to whiplash in patients with mild traumatic brain injury (mTBI).

**Methods:** Thirty-one patients with mTBI and 21 healthy control subjects were recruited for this study. The balance error scoring system (BESS) was used for PCA assessment. Based on their whiplash history, the patients were classified into two groups: group A—mTBI with whiplash injury; group B—mTBI without whiplash injury. Fractional anisotropy (FA), apparent diffusion coefficient (ADC), and tract volume (TV) values were estimated for the reconstructed CRTs in all subjects.

**Results:** Significant differences were observed among the total BESS scores of patient groups A and B and the control group (*p* < 0.05). The patient group A BESS score was significantly higher than that of patient group B, and that of the patient group B was significantly higher than that of the control group. No significant differences were detected among the FA and ADC values of the CRTs of the two patient groups and the control group (*p* > 0.05). However, the TV values of the CRT did reveal significant differences; the TV of patient group A was significantly lower than those of patient group B and the control group, and that of patient group B was significantly lower than that of the control group (*p* < 0.05).

**Conclusions:** We observed greater CRT injury severity and PCA impairment in mTBI patients with whiplash than in mTBI patients without whiplash. The results indicate that whiplash might lead to a greater level of severity in axonal injuries in mTBI patients.

## Introduction

Traumatic brain injury (TBI) can be caused by a sudden impact or an acceleration–deceleration trauma to the head (1). TBI can be classified as mild, moderate, or severe based on the level of injury severity with mild TBI (mTBI) comprising ~75–90% of all TBI (2, 3). Whiplash is a bony and/or soft tissue injury resulting from acceleration–deceleration energy transfers in the neck (4). Patients with whiplash often complain of cerebral symptoms suggestive of brain injury, and previous studies have reported evidence that indicates the presence of brain injury in patients with whiplash (5–12). Therefore, whiplash has been considered as a pathogenic mechanism of mTBI (13).

Presence of postural control means the subject has the ability to maintain postural orientation in response to perturbations generated from internal or external sources (14). Impairment of postural control ability (PCA) is a common clinical manifestation following mTBI and/or whiplash (14–20). Previous studies have suggested that mTBI due to whiplash might be associated with greater impairment of PCA than that in patients with mTBI due to other causes (16, 17). The corticoreticulospinal tract (CRT), one of the extrapyramiidal motor pathways in the human brain, mainly mediates movements of proximal and axial muscles (21, 22). As a result, the CRT is involved in postural control responses to perturbations generated from internal or external sources (14, 21, 22).

The introduction of diffusion tensor tractography (DTT), a three-dimensional (3D) modeling process that uses diffusion tensor imaging (DTI) data, has enabled 3D reconstruction and estimation of neural tracts, including the CRT (23). The main advantage of DTT over DTI is that the entire neural tract can be evaluated in terms of DTT parameters. Among the various DTT parameters that can be examined, fractional anisotropy (FA), apparent diffusion coefficient (ADC), and tract volume (TV) parameters are the most commonly used (24–26). The FA value, which indicates the degree of directionality of water diffusion, is used to assess the degree of tract directionality whereas the ADC value indicates the magnitude of the water diffusion (24, 25). Therefore, the values of FA and ADC may be used to suggest the microstructural integrity of white matter microstructures, such as axons, myelin, and microtubules (24, 25, 27, 28). In contrast, the TV value indicates the number of voxels (values on a regular grid within a 3D space) that are included in a neural tract (26). A decrement in TV value without a change in the values of FA and ADC of a neural tract indicates a decrement in the number of neural fibers of that tract without a change in the microstructural integrity of the tract (24–26). Therefore, decreases in the values of the FA and TV, as well as increases in the values of the ADC, indicate the presence of neural injury (24–28). A few studies have used DTT to demonstrate CRT injuries in patients with mTBI (29–31). Among these studies, one demonstrated an association between CRT injury and PCA (31). However, no study has reported on the relationship between CRT injury and PCA impairment according to whiplash status in mTBI patients.

For the present study, we hypothesized that PCA impairment and CRT injury would differ according to whiplash presence in mTBI patients. Therefore, we investigated differences in PCA and CRT injuries in mTBI patients with and without a history of whiplash.

## Methods

### Subjects

A total of 39 consecutive patients with mTBI (18 males, 21 females; mean age 46.20 ± 10.92 years; age range 21–64 years; from January 2016 to December 2018) who visited the rehabilitation department of a university hospital and 20 age- and sex-matched normal control subjects (12 male, 9 female; mean age 45.1 ± 8.93 years; age range 30–64 years) with no history of neurologic/psychiatric disease were recruited for this study (Table 1).

**Table 1:** Demographic and clinical data for the patient and control groups.

	**Patient group A (*n* = 19)**	**Patient group B (*n* = 20)**	**Control group (*n* = 21)**
Gender (male:female)	8:11	10:10	12:9
Mean age, years^a^	49.47 (±10.55)	43.10 (±10.58)	45.10 (±8.93)
Mild traumatic brain injury causal mechanism	Motor vehicle accident (100%)	Motor vehicle accident (85%)	–
		Falling (10%)	
		Direct head trauma (5%)	

a *Values represent mean (± standard deviation)*.

^*^Significantly different among patient A, B, and control at p <0.05.

Inclusion criteria for the 39 patients were: (1) more than 2 weeks had elapsed after mTBI onset, (2) at least one of the following: (i) any period of loss of consciousness; (ii) any loss of memory for events immediately before or after the accident; (iii) any alteration in mental state at the time of the accident (e.g., feeling dazed, disoriented, or confused); or (iv) any focal neurological deficit(s) that may or may not be transient but in which the severity of the injury does not exceed the following: loss of consciousness of approximately 30 min or less; after 30 min post-onset, an initial Glasgow Coma Scale of 13–15; and post-traumatic amnesia (PTA) of <24 h (13, 32, 33), (3) no specific lesion observed on brain magnetic resonance imaging (MRI; T1-weighted, T2-weighted, and fluid-attenuated inversion recovery images), (4) age at the time of head trauma >18 years, (5) an abnormal score on the balance error scoring system (BESS) (cut-off score ≥ 14, range: 0–60; a higher value means more severe postural impairment) (34), and (6) no previous history of neurologic/psychiatric disease. Patients were excluded if impairments could have been due to drugs, alcohol, medications, other injuries, treatment for other injuries, other problems, or the result of a penetrating craniocerebral injury.

We divided the patient group into two groups based on the patient's history of whiplash (indicated by the presence of flexion-hyperextension, lateral flexion, or rotation injury of the head and neck following a motor vehicle collision). Nineteen patients were placed in patient group A (mTBI with whiplash injury; injury causes: motor vehicle accidents, 100%) and 20 patients were assigned to patient group B [mTBI without whiplash injury; injury causes: motor vehicle accidents, 17 (85%); falling, 2 (10%); direct head trauma, 1 (5%)] (Table 1). No significant differences in age or sex compositions were detected between patient groups A and B or between the patient and control groups (*p* > 05).

This was a retrospective study, and the study was carried out in accordance with the recommendations of “The CARE of guidelines” with written informed consent obtained from all subjects. The patient provided written informed consent in accordance with the Declaration of Helsinki, and the study protocol was approved by the Institutional Review Board of the Yeungnam University hospital.

### Clinical Evaluation

BESS was used for the evaluation of PCA at the time of DTI scanning. Evaluation using BESS has been shown to provide high validity and reliability in normal subjects (35). In addition, BESS can be used to assess the effects of a mild head injury on PCA in the absence of expensive, sophisticated PCA assessment tools (35, 36). Determination of a BESS score requires the subject to stand unsupported with their eyes closed under six conditions involving a combination of two surface types (firm and balance foam) and three stances (double-limb, single-limb, and tandem). In this study, the balance foam (Airex, Sins, Switzerland) was a 6 cm thick piece of medium-density foam (dimensions 50 × 41 cm, density 55 kg/cm^3^, tensile strength 260 kPa, elongation to breaking 180%). The non-dominant leg was used as the stance limb during the single-leg trials and it was placed in the rear position during tandem stance trials. The preferred leg to use while kicking a ball was defined as the dominant leg. The order of BESS testing conditions of each individual was randomized by subject and session, with each test lasting 20 s (37). For each test, subjects were asked to assume the required stance by positioning their hands on their iliac crests. A test commenced upon eye closure by the subject. Subjects were instructed to try not to lose their balance but, if needed, make any necessary adjustments and return to the testing position as quickly as possible. Test performance was scored by the addition of one error point for each error committed. An error was assigned when any of the following occurred: (1) lifting hands off the iliac crests; (2) opening the eyes; (3) stepping, stumbling, or falling; (4) moving the hip by more than 30 degrees of flexion or abduction; (5) lifting the forefoot or heel; or (6) remaining out of the testing position for more than 5 s (37). The maximum number of errors for a single stance condition was 10. If the subject could not maintain a stance position for longer than 5 s, subjects were assigned the maximum score for that position. The numbers of errors for each trial were added to obtain a total score (0–60; lower scores indicated better balance) (35).

### Diffusion Tensor Imaging and Fiber Tracking

The DTI data obtained for this study were acquired at an average of 14.0 ± 26.2 months after the onset of TBI by using a 1.5 T Philips Gyroscan Intera scanner (Philips, Best, Netherlands) equipped with a Synergy-L sensitivity encoding (SENSE) head coil in order to obtain single-shot, spin-echo, planar-imaging pulse sequences. For each of the 32 non-colinear diffusion sensitizing gradients, 60 contiguous slices were acquired parallel to the anterior commissure–posterior commissure line. Imaging parameters were as follows: acquisition matrix = 96 × 96, reconstructed to matrix = 192 × 192, field of view = 240 × 240 mm, repetition time = 10,398 ms, time to echo = 72 ms, parallel imaging reduction factor (SENSE factor) = 2, EPI factor = 59, and b = 1,000 s/mm^2^, number of excitations = 1, and thickness = 2.5 mm. Eddy current-induced image distortions were removed by using affine multiscale two-dimensional registration as provided within the Oxford Centre for Functional Magnetic Resonance Imaging of Brain Software Library (www.fmrib.ox.ac.uk/fsl). DTI-Studio software (CMRM; Johns Hopkins Medical Institute, Baltimore, MD, USA) was used for evaluation of the CRT. The CRT was reconstructed based on fibers passing through two regions of interest (ROIs) on the DTI color map. The first ROI was placed at the reticular formation of the medulla. The second ROI was placed at the tegmentum of the midbrain. Termination criteria used for fiber tracking were a fractional anisotropy (FA) value of <0.2 and an angle of <60 degrees (23). The FA, ADC, and TV values for the DTT-reconstructed CRT were obtained for both hemispheres.

### Statistical Analysis

Statistical analysis was performed by using SPSS 21.0 for Windows (SPSS, Chicago, IL, USA). One-way analysis of variance with Fisher's least significant difference *post hoc* test was performed to determine the significance of differences in BESS scores, DTT parameters (FA, ADC, and TV) of the CRT, and age distribution among patient groups A and B and the control group. The chi-squared test was used to examine gender-based differences between patient groups A and B and the control group.

## Results

Comparisons of BESS scores and DTT parameter results for the CRTs of the patient and control groups are summarized in Table 2. The mean total BESS score for patient group A was significantly higher than those of patient group B and the control group; furthermore, the mean total BESS score for patient group B was significantly higher than that of the control group (*p* < 0.05).

**Table 2:** Clinical data and diffusion tensor imaging parameters for the corticoreticulospinal tracts of patient groups A and B and the control group.

	**Patient group A (*n* = 19)**	**Patient group B (*n* = 20)**	**Control group (*n* = 21)**	**F**	***P***
BESS	38.73 (±13.56)^a^	20.42 (±6.23)^b^	10.81 (±3.98)^c^	46.13	<0.00
FA	0.48 (±0.03)	0.48 (±0.03)	0.48 (±0.01)	0.08	0.92
ADC	0.80 (±0.09)	0.79 (±0.05)	0.78 (±0.03)	0.51	0.61
TV	551.63 (±281.74)^a^	870.05 (±327.16)^b^	1,368.52 (±264.86)^c^	39.94	<0.00

*Values represent mean (± standard deviation); BESS, balance error scoring system; CRT, corticoreticulospinal tract; FA, fractional anisotropy; ADC, apparent diffusion coefficient; TV, tract volume; One-way ANOVA and the Scheffe post hoc test were used for comparison of diffusion tensor parameters among patient groups A and B and the control group*.

*Scheffe test result: a < b < c*.

**Figure 1:**
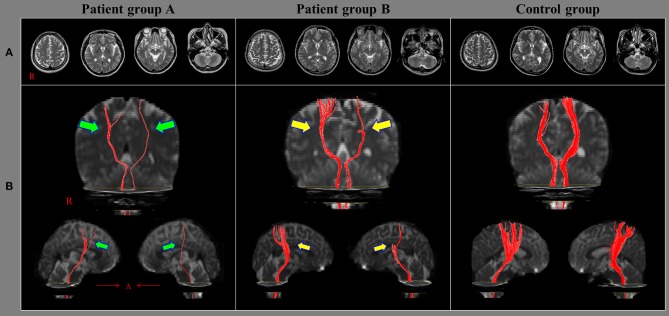
Results of diffusion tensor tractography (DTT) of the corticoreticulospinal tract (CRT). **(A)** T2-weighted brain magnetic resonance images obtained at the time of diffusion tensor imaging in representative subjects of patient group A (51-year-old female), patient group B (49-year-old male), and the control group (56-year-old male); none of the images show an abnormality. **(B)** Results of DTT of the CRT: the CRTs in the group A patient (green arrows) are narrower on both sides than those of the group B patient (yellow arrows). Moreover, the CRTs of the group B patient (yellow arrows) are narrower on both sides than those of the control group patient.

No significant differences were observed in the mean FA and ADC values of the CRTs of patient groups A and B and those of the control group (*p* > 0.05). However, the mean TV value of the CRT of patient group A was significantly lower than those of patient group B and the control group; moreover, the mean TV value of the CRT of patient group B was significantly lower than that of the control group (*p* < 0.05) (Figure 1).

## Discussion

In this study, we investigated differences in PCA and CRT injury among mTBI patients with and without a history of whiplash injury. The results may be summarized as follows: (1) PCA, as determined by BESS testing, became increasingly worse in the following order: control group, patient group B (mTBI without whiplash), and patient group A (mTBI with whiplash); (2) TV values for the CRTs of the three study groups decreased in the same order, but there were no significant differences in FA and ADC values among the groups.

In the clinical evaluation of a patient's PCA, a high BESS score indicates a high level of impairment of PCA. In this study, the most severe PCA impairment was observed in mTBI patients that experienced whiplash with a lower level of impairment observed in mTBI patients that did not have a history of whiplash. These results indicate that whiplash can produce a more severe effect on the PCA of mTBI patients than that from mTBI alone. This result supports those in previous studies which showed that PCA impairment was greater in mTBI patients with a whiplash injury (16, 17).

In 2011, Nacci et al. assessed shifting of the centers of gravity in whiplash patients with and without mTBI and reported that whiplash patients with mTBI showed greater center of gravity shifting than that of whiplash patients without mTBI (16). In 2016, Gandelman-Marton et al. investigated differences in body weight distribution between mTBI patients with and without whiplash and reported that there was a greater change in body weight distribution in mTBI patients with whiplash than in mTBI patients without whiplash (17).

Among the various DTT parameters that can be examined, FA, ADC, and TV are the most commonly used when evaluating the state of neural tracts in patients with brain injury (24–26). Our results showed a decrement in TV value without a change in the FA and ADC values of the CRT, indicating a decrease in the number of neural fibers in the CRT without a change in its microstructural integrity (24–26). As a result, our TV results indicate the presence of CRT injuries in both patient groups, but the CRT injuries exhibited greater severity in mTBI patients with whiplash than that in those without whiplash. The association of CRT injury with PCA appears to be related to the role of the CRT, which, by mediating movements of proximal and axial muscles, is involved in postural control responses to perturbations generated from internal or external sources (14, 21, 22). Because no brain lesions were detected on conventional brain MRI, traumatic axonal injury was considered the most likely pathogenetic mechanism for the CRT injuries identified in our patient groups (38–40). Based on our results, we conclude that the indirect acceleration–deceleration forces transmitted to the whole brain during whiplash might contribute to increasing the severity of axonal injury to the CRT to a level greater than that in mTBI patients with CRT injuries due to other causes (4, 12).

Since the introduction of DTI, a few studies have demonstrated CRT injuries in patients with mTBI (29–31). Two studies reported on patients with mTBI who had more severe weakness in their proximal joints than in their distal joints, which was indicated to be a consequence of CRT injury (29, 30). Jang et al. (31) evaluated PCA and CRT status in 25 mTBI patients and demonstrated that PCA impairment is associated with the presence of CRT injury. Nonetheless, to our best knowledge, this is the first original study to demonstrate that whiplash can lead to increased axonal injury severity in the CRT of mTBI patients.

However, there are some limitations to this study that should be considered. First, DTT analysis is operator-dependent, and regions of fiber complexity and crossing can prevent full reflection of the underlying fiber architecture (41). Second, because our patients were recruited from those who visited the rehabilitation department of a university hospital, there was a possibility that patients with a distinct level of severity of clinical manifestations might be included in this study; thus, our results may not be comparable to those from a broader population of mTBI patients with whiplash injury. Third, we did not perform a clinical examination of the impairments to cervical muscle function due to whiplash; variation in which can affect the CRT (42). Hence, we could not rule out the effect of cervical muscles on our results. Fourth, we recruited patients with chronic stage mTBI (i.e., more than 2 weeks after onset) and the average duration to DTI after onset was heterogeneous (14.0 ± 26.2 months) after TBI onset. As a result, recovery or degeneration of the CRT injuries during that interval may have influenced the results (43–45).

In conclusion, we investigated the association of whiplash with PCA and CRT injury in mTBI patients and observed that CRT injuries and PCA impairments were more severe in mTBI patients with whiplash than in those without whiplash. Our results suggest that whiplash might lead to more severe axonal injury in mTBI. Further studies on the effect of whiplash on other neural tracts are warranted.

## Disclosure

Financial disclosure statements have been obtained, and no conflicts of interest have been reported by the authors or by any individuals in control of the content of this article.

## Data Availability Statement

The datasets generated for this study are available on request to the corresponding author.

## Ethics Statement

This study was carried out in accordance with the recommendations of The CARE of guidelines with written informed consent from all subjects. The patient signed a written informed consent in accordance with the Declaration of Helsinki, and the study protocol was approved by the Institutional Review Board of the Yeungnam University hospital.

## Author Contributions

SJ and YK were involved in manuscript development, funding, data acquisition, and manuscript writing. SY and JK helped in conceiving, designing the study, manuscript development, and manuscript writing.

### Conflict of Interest

The authors declare that the research was conducted in the absence of any commercial or financial relationships that could be construed as a potential conflict of interest.
